# Hierarchically Structured V_2_O_5_/PANI Heterostructures for Room-Temperature Ammonia Sensing

**DOI:** 10.3390/s26165300

**Published:** 2026-08-21

**Authors:** Chunmei Shangguan, Anan Xu, Fang Wang, Ying Li, Jiao Jia, Zhenchen Liu

**Affiliations:** 1 Institute of Unmanned System, Bei Hang University, Beijing 100191, China; shangguanchunmei@126.com (C.S.); buaaairforce@buaa.edu.cn (F.W.); 2 School of Instrument Science and Opto-Electronics Engineering, Beijing Information Science and Technology University, Beijing 100192, China; liyingbistu0010@163.com; 3 School of Aviation, Bei Hang University, Beijing 100191, China; jiajiao@buaa.edu.cn

**Keywords:** ammonia sensor, V_2_O_5_/PANI, room temperature, heterostructure, hierarchical structure

## Abstract

**Highlights:**

- Hierarchical V_2_O_5_/PANI heterostructures are synthesized via coprecipitation and in situ polymerization.
- The interpenetrated architecture suppresses aggregation and boosts interfacial sites.
- PAV20 achieves a 2.5–fold response to 10 ppm NH3 at room temperature.
- Performance enhancement originates from morphology and band alignment synergy.

**Abstract:**

Ammonia, a toxic and volatile pollutant commonly found in chemical industrial environments, requires reliable real-time detection to ensure industrial safety and effective environmental monitoring. Conventional gas sensors typically operate at elevated temperatures, resulting in high power consumption. Moreover, pure metal oxides and conductive polymers often suffer from significant aggregation and exhibit suboptimal sensing performance under ambient conditions, limiting their practical applications. In this study, hierarchical porous V_2_O_5_/PANI composites were synthesized via a straightforward one-step coprecipitation method combined with in situ polymerization. The interlaced architecture of polyaniline (PANI) and vanadium pentoxide (V_2_O_5_) effectively reduces structural aggregation and increases the availability of surface active sites. Furthermore, the synergistic interaction at the bi-phase interface significantly enhances charge carrier transport, leading to improved ammonia-sensing capabilities at room temperature. Notably, the composite containing 20% V_2_O_5_ demonstrated superior response, selectivity, and reproducibility toward 10 ppm NH_3_. Due to its simple fabrication process and room-temperature operation without external heating, the developed V_2_O_5_/PANI composite sensor holds significant potential for practical applications in low-concentration ammonia detection under ambient conditions.

## 1. Introduction

Ammonia (NH_3_) is extensively utilized in chemical manufacturing processes [1]. Its leakage can generate toxic and explosive gas mixtures, presenting significant safety risks that necessitate stringent oversight in chemical industrial zones. Traditional methods, including manual inspections and fixed-point monitoring, are limited by substantial blind spots and delayed detection, thereby underscoring the urgent need for innovative ammonia-sensing elements characterized by high selectivity and low power consumption. Metal oxide semiconductors (MOSs) exhibit high sensitivity and excellent chemical stability, making them widely employed in industrial gas monitoring applications [2,3,4,5]. Nevertheless, conventional MOS gas sensors require continuous high-temperature heating to maintain stable operation. The integrated heating component poses a potential ignition hazard, which could lead to explosion incidents in flammable and explosive chemical environments. Additionally, the constant heating required by traditional MOS sensors increases power consumption and poses ignition hazards in flammable gas environments. These drawbacks fundamentally hinder the development of compact, portable monitoring devices that can operate safely without built-in heating elements.

Microscopic morphology plays a fundamental role in governing the gas-sensing performance of functional materials [6]. Materials exhibiting hierarchically porous architectures facilitate the formation of continuous gas diffusion pathways, thereby significantly enhancing the mass transfer kinetics of target gas molecules. Concurrently, the increased specific surface area exposes a multitude of intrinsic oxygen vacancy defects, with these unsaturated coordination sites acting as primary active centers that promote interfacial gas adsorption and redox reactions [7,8]. To overcome the inherent performance limitations of conventional MOSs, extensive research has been dedicated to the precise nanostructural engineering of gas-sensing materials [9,10]. Leveraging the synergistic effects of pore channel transport and surface defect activation, hierarchically structured MOSs have emerged as highly promising substrates for gas-sensing applications. In recent years, numerous hybrid heterostructures, formed by integrating MOSs with one-dimensional or two-dimensional nanomaterials, have been developed to further enhance sensing performance [11,12,13]. Despite the advances achieved through morphological optimization, challenges such as high operating temperatures and limited gas selectivity continue to impede the practical deployment of MOS-based sensors. The dissociative adsorption of oxygen molecules at low temperatures necessitates overcoming a substantial activation energy barrier, thereby requiring elevated temperatures to generate sufficient active oxygen species [14]. Moreover, single-component MOSs typically exhibit broad cross-sensitivity to various gases, which hinders their ability to selectively and accurately identify target gases in complex multi-component environments.

Among various resistive MOS sensing platforms, vanadium-based oxides have emerged as prominent research subjects for NH_3_ sensors, owing to their reversible multivalent transition characteristics and distinctive optoelectronic transport properties [15,16,17,18]. V_2_O_5_, recognized as the thermodynamically most stable highest-valence phase of vanadium oxides, exhibits a characteristic layered van der Waals crystal structure [19]. The interlayer spaces in V_2_O_5_ serve as advantageous intercalation pathways for gas molecules, conferring excellent thermal stability, high catalytic oxidation activity, and wide-bandgap semiconductor properties [20]. Theoretically, V_2_O_5_ is suitable for detecting a range of gases, including NH_3_, NO_2_, low-carbon alcohols, amines, and hydrocarbon fuels, and it can physically pre-adsorb NH_3_ and O_2_ molecules at ambient temperature. However, pure-phase V_2_O_5_ presents notable intrinsic limitations. Its wide bandgap results in an extremely low density of intrinsic charge carriers, and the limited efficiency of reversible V^5+^/V^4+^ redox charge transfer leads to suboptimal sensing response amplitudes [21]. Furthermore, pristine V_2_O_5_ lacks specific gas recognition sites and is prone to significant cross-sensitivity in mixed-gas environments, thereby hindering its ability to selectively detect trace amounts of NH_3_. Consequently, strategic modifications are imperative to enhance its gas-sensing performance and practical applicability.

Organic conductive polymers exhibit sensing mechanisms that complement those of MOSs and have garnered significant research interest due to their adjustable proton-doping conductivity, mild synthesis conditions, excellent mechanical flexibility, and capability for passive detection at ambient temperature [22,23]. Polyaniline (PANI), a prototypical p-type conductive polymer, facilitates efficient conduction at room temperature through polaron and bi-polaron charge transport mechanisms [24]. Owing to its cost-effectiveness and environmental compatibility, PANI can engage in specific proton exchange reactions with NH_3_ molecular structures [25], indicating substantial potential for the detection of trace amounts of NH_3_ [26,27,28]. In comparison to zero-dimensional granular PANI, one-dimensional PANI nanofibers form continuous and interconnected conductive networks that effectively lower charge transport barriers. Additionally, the nanofibrous morphology provides a high specific surface area, which increases the number of interfacial active sites, shortens gas diffusion pathways, and enhances the efficiency of interfacial electron exchange between the sensing material and target gases. However, pure PANI is still limited by intrinsic drawbacks such as inadequate long-term stability, restricted sensitivity, and slow response and recovery kinetics, which significantly constrain its use as a standalone material for high-precision and long-duration NH_3_-sensing applications.

Leveraging the complementary advantages of V_2_O_5_ and PANI in sensing capabilities and operational mechanisms, this study develops a hierarchically structured V_2_O_5_/PANI heterojunction to synergistically integrate the strengths of both materials. The interfacial band bending effect enables precise modulation of the carrier depletion layer width, effectively combining the high catalytic activity associated with oxygen vacancies in V_2_O_5_ and the room-temperature operation, low power consumption, and high NH_3_ selectivity characteristics of PANI [29]. This approach addresses the inherent performance limitations of individual V_2_O_5_ and PANI materials from a microscopic mechanistic perspective. In this investigation, V_2_O_5_ nanosheets were synthesized via a straightforward coprecipitation method, followed by the fabrication of V_2_O_5_/PANI composite sensing materials through in situ chemical oxidative polymerization. By carefully tuning the compositional ratio of V_2_O_5_ to PANI, a hierarchical architecture comprising V_2_O_5_ nanosheets and PANI nanofibers with a high specific surface area was successfully constructed. The deliberately engineered heterostructure induces pronounced interfacial synergistic coupling effects, enhancing the utilization efficiency of active sites and charge transport properties, while optimizing the adsorption, desorption, and interfacial redox reaction kinetics of NH_3_. Owing to the combined enhancements from structural design and interfacial mechanisms, the optimized V_2_O_5_/PANI composite demonstrates effective detection of trace-level NH_3_ at ambient temperature. This work offers a viable strategy for structural modulation in the design and synthesis of high-performance room-temperature ammonia sensors, with potential applications in industrial safety monitoring.

## 2. Materials and Methods

### 2.1. Synthesis

V_2_O_5_ nanomaterials were fabricated via a facile one-step coprecipitation strategy. First, 0.5 mmol of NH_4_VO_3_ was dissolved in 50 mL of ultrapure water under magnetic stirring to form a precursor solution. Subsequently, 5.5 mL of 1 M HCl solution was added dropwise under vigorous stirring to generate orange-red precipitates. The precipitates were harvested and repeatedly washed until the filtrate was neutral. After drying at 70 °C overnight, the powder was loaded into a ceramic boat and calcined at 500 °C in ambient air using a muffle furnace, yielding dark-orange V_2_O_5_ particles.

Hierarchical V_2_O_5_/PANI composite sensitive structures were further fabricated through an in situ chemical oxidative polymerization strategy. The entire polymerization reaction was carried out in an ice-water bath. First, 1 mmol of ammonium persulfate (APS) was dissolved in 10 mL of 1 M hydrochloric acid solution and stirred continuously for 30 min to obtain a uniform initiator solution. Meanwhile, a series of V_2_O_5_ nanoparticles with different molar doping ratios (0, 5%, 10%, 20%, 30%, 40%, and 50%, relative to aniline monomers) were dispersed in 30 mL of 1 M hydrochloric acid solution. After stirring for 30 min in an ice-water bath to form a stable dispersion, 1 mmol of purified aniline monomer was added to the dispersion to prepare the composite precursor solution. Thereafter, the pre-configured APS solution was slowly dropped into the precursor solution, and the mixture was continuously stirred for 2 h in an ice-water bath, followed by static standing to obtain a dark green-black product solution. The resulting products were collected by centrifugation and thoroughly washed to neutrality. After constant-temperature drying at 70 °C overnight, a series of V_2_O_5_/PANI composites were successfully synthesized. According to the different molar doping ratios of V_2_O_5_, the composites were labeled as PAV0, PAV5, PAV10, PAV20, PAV30, PAV40, and PAV50.

### 2.2. Characterization

Scanning electron microscope (SEM) images of the samples were obtained by using a ZEISS Gemini 300 (ZEISS, Oberkochen, Germany). Elemental mapping was conducted via SEM with energy-dispersive X-ray spectroscopy (EDS). The crystal structure of the samples was evaluated using X-ray diffraction (XRD) analysis carried out on a Rigaku D/Max 2550 (Rigaku Corporation, Tokyo, Japan) equipped with a Cu Kα X-ray source (λ = 1.5418 Å). X-ray photoelectron spectroscopy (XPS) was conducted with a Thermo Scientific K-Alpha spectrometer (Thermo Fisher Scientific, Waltham, MA, USA) equipped with a monochromated Al Kα X-ray source (1486.6 eV).

### 2.3. Sensor Fabrication and Measurement

To fabricate gas-sensing devices, the as-synthesized powders were formulated into homogeneous slurries, which were evenly coated on the surface of preprocessed alumina ceramic tubes (length: 4 mm; outer diameter: 1.35 mm; wall thickness: 0.35 mm) to construct functional thick films with an electrode spacing of approximately 3 mm. The obtained sensing film had a thickness on the order of 200–500 μm, sufficient to fully cover the ceramic tube surface. Then, the devices were dried overnight in ambient air at room temperature to enhance the operational stability of the sensors.

**Figure 1:**
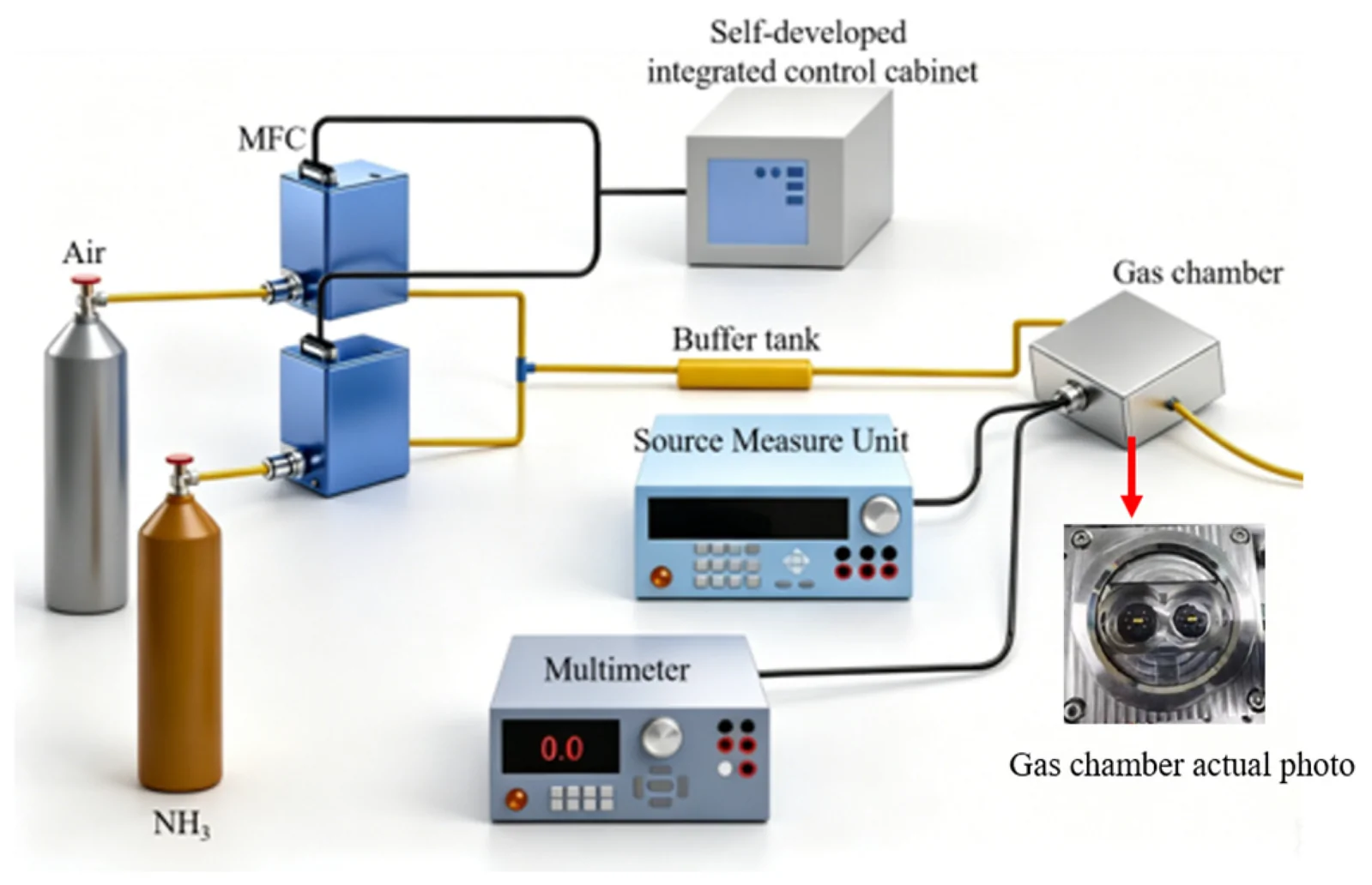
Schematic of the dynamic testing device.

Gas-sensing measurements were performed using a custom-built test chamber with an internal volume of approximately 30 mL. The sensor was mounted directly onto the circuit board integrated within the test chamber. A schematic diagram of the test apparatus is shown in Figure 1. All measurements were conducted in a clean room with constant-temperature and -humidity conditions (23 ± 2 °C, 25 ± 5% RH). Gas mixtures of desired concentrations were prepared using mass flow controllers (MFCs) connected through a control cabinet, which allowed precise regulation of the flow rates of the test gas (NH_3_/air standard gas) and the dilution gas (clean dry air), thereby controlling the target gas concentration. The test gas and dilution gas were first mixed in a buffer tank before being introduced into the test chamber. The buffer tank served a dual purpose: it ensured thorough and uniform mixing of the gas streams, and it attenuated pressure fluctuations to minimize airflow disturbance on the sensor surface. The test chamber was equipped with gas inlet and outlet ports on opposite sides, through which the flowing test gas of a predetermined concentration was continuously delivered. Up to two sensor devices could be tested simultaneously inside the chamber. A total gas flow rate of 400 sccm was maintained throughout all measurements. Each measurement cycle consisted of a gas exposure phase followed by a purge phase in clean air. The exposure duration was set to approximately 20 min for all tested concentrations to ensure consistent comparison conditions. For the concentration-dependent characterization, the purging time was controlled within 20 min for low ammonia concentrations (≤2 ppm), while extended purging of approximately 25–35 min was employed for higher concentrations (5–20 ppm) due to the stronger adsorption and slower desorption kinetics at elevated loading levels. For the long-term stability test, the exposure time was reduced to approximately 15 min, with an extended recovery period of 50 min in clean air to facilitate baseline recovery between consecutive cycles.

The resistance of the sensor was measured directly using a Keithley 2400 source meter (Keithley Instruments, Cleveland, OH, USA) without applying any external bias voltage. For data processing, we define three resistance parameters: R_0_ refers to the initial baseline resistance of the sensor at the very beginning of the whole test procedure; R_a_ stands for the real-time baseline resistance in air recorded after stabilization and before each test gas injection in each independent cycle; and R_g_ represents the stable resistance value after the test gas is introduced, defined as the point at which the resistance change rate falls below 1% per 2 min. The sensing response of the sensor is calculated as R_g_/R_a_.

## 3. Results

### 3.1. Material Characterization Analysis

**Figure 2:**
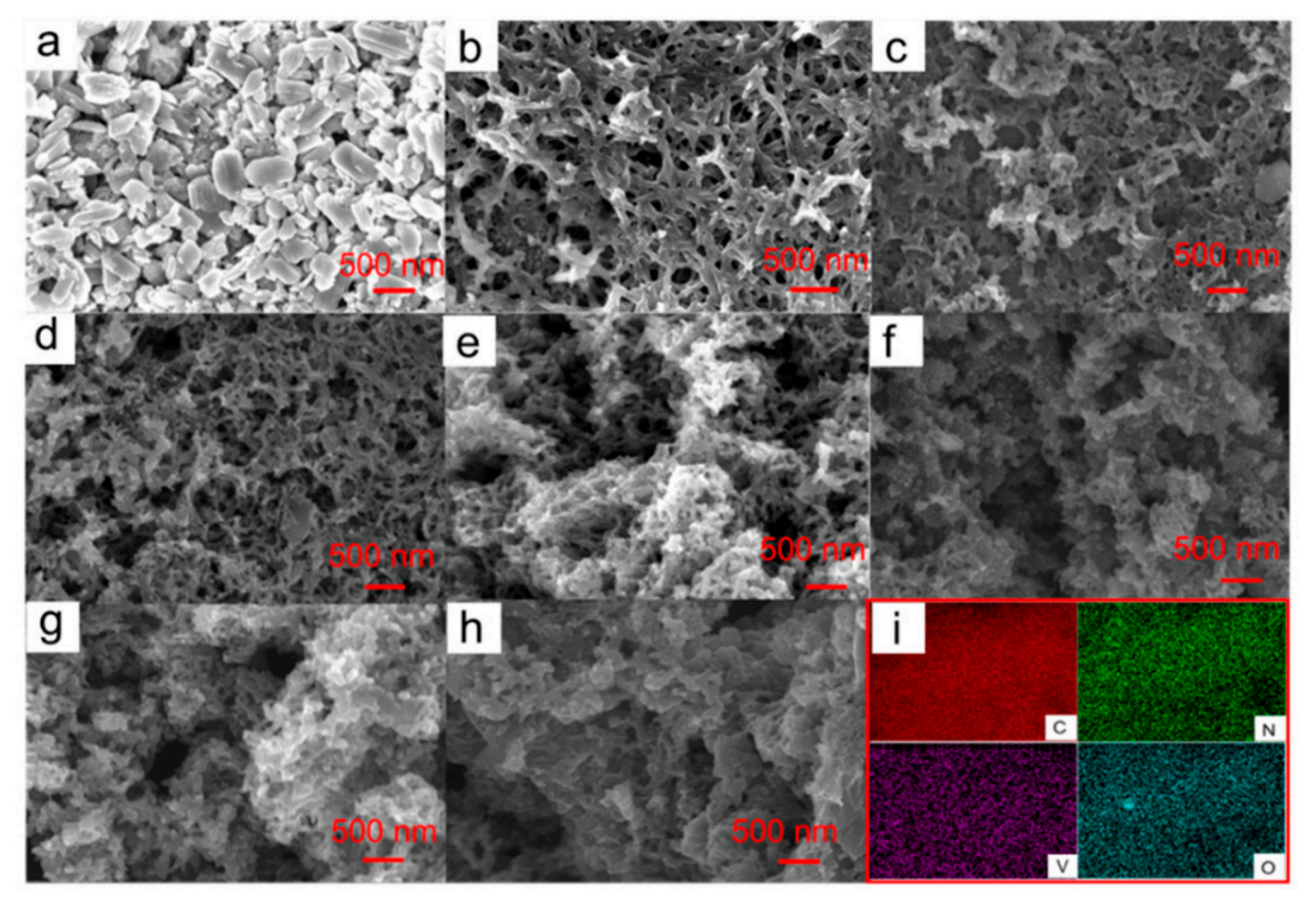
(**a**–**h**) SEM images of synthesized materials: (**a**) V_2_O_5_, (**b**) PAV0, (**c**) PAV5, (**d**) PAV10, (**e**) PAV20, (**f**) PAV30, (**g**) PAV40, and (**h**) PAV50. (**i**) EDS element mapping images of C, N, V, and O for PAV20.

SEM was utilized to characterize the micromorphology of all as-synthesized specimens, including pure V_2_O_5_, pure PANI, and a series of V_2_O_5_/PANI composites. The corresponding characterization results are displayed in Figure 2. Figure 2a shows the SEM graph of pure V_2_O_5_, which possesses a regular stacked sheet-like morphology with an average sheet size of around 200 nm. Extensive stacking of these sheets results in a compact bulk structure with limited inner pores. Figure 2b presents the micromorphology of pure PANI, where uniform and continuous nanofibers intertwine with each other to form a basic three-dimensional network skeleton. Figure 2c–h are the SEM images of V_2_O_5_/PANI composites with V_2_O_5_ molar doping fractions of 5%, 10%, 20%, 30%, 40%, and 50%, respectively. Variations in doping ratios cause remarkable differences in composite micromorphology. At low V_2_O_5_ doping levels (5% and 10%), the overall morphology of the composites resembles that of pure PANI nanofibers, and V_2_O_5_ sheets are homogeneously dispersed inside the fiber network without obvious agglomeration. Nevertheless, the low loading content of V_2_O_5_ fails to fully adjust the microscopic structure of the composite. When the V_2_O_5_ doping fraction reaches 20%, appropriate amounts of V_2_O_5_ sheets are evenly attached and embedded on the surface and in the gaps of the PANI fiber network. The interconnected three-dimensional fiber skeleton of PANI is completely retained, and uniformly distributed V_2_O_5_ simultaneously creates abundant pore structures, with no structural defects such as particle agglomeration or sheet stacking observed in the whole sample. The hierarchical porous architecture observed in PAV20 provides abundant gas-accessible interfaces for NH_3_ interaction. The V_2_O_5_ nanoparticles uniformly decorated on the PANI backbone create interparticle voids and a porous network that facilitate rapid gas diffusion. Such hierarchical V_2_O_5_/PANI composites have been reported to exhibit significantly enhanced specific surface areas compared to bare V_2_O_5_ and PANI [30,31,32], which contributes to the improved sensing performance observed in this work. Once the V_2_O_5_ doping fraction rises to 30% or higher, severe agglomeration and stacking of V_2_O_5_ sheets take place. This behavior not only reduces the effective specific surface area and blocks internal pore channels but also compresses and fractures the continuous PANI fiber network, ultimately leading to a densified composite structure. EDS elemental mapping measurement was carried out on the PAV20 sample to visually confirm the dispersion and composite state of the two phases, and the results are shown in Figure 2i. The N elements derived from PANI and the V elements derived from V_2_O_5_ are uniformly scattered across the observation area, without local elemental enrichment, particle agglomeration, or phase separation. This result verifies that homogeneous blending and composite construction between V_2_O_5_ and PANI can be realized at a doping fraction of 20%.

**Figure 3:**
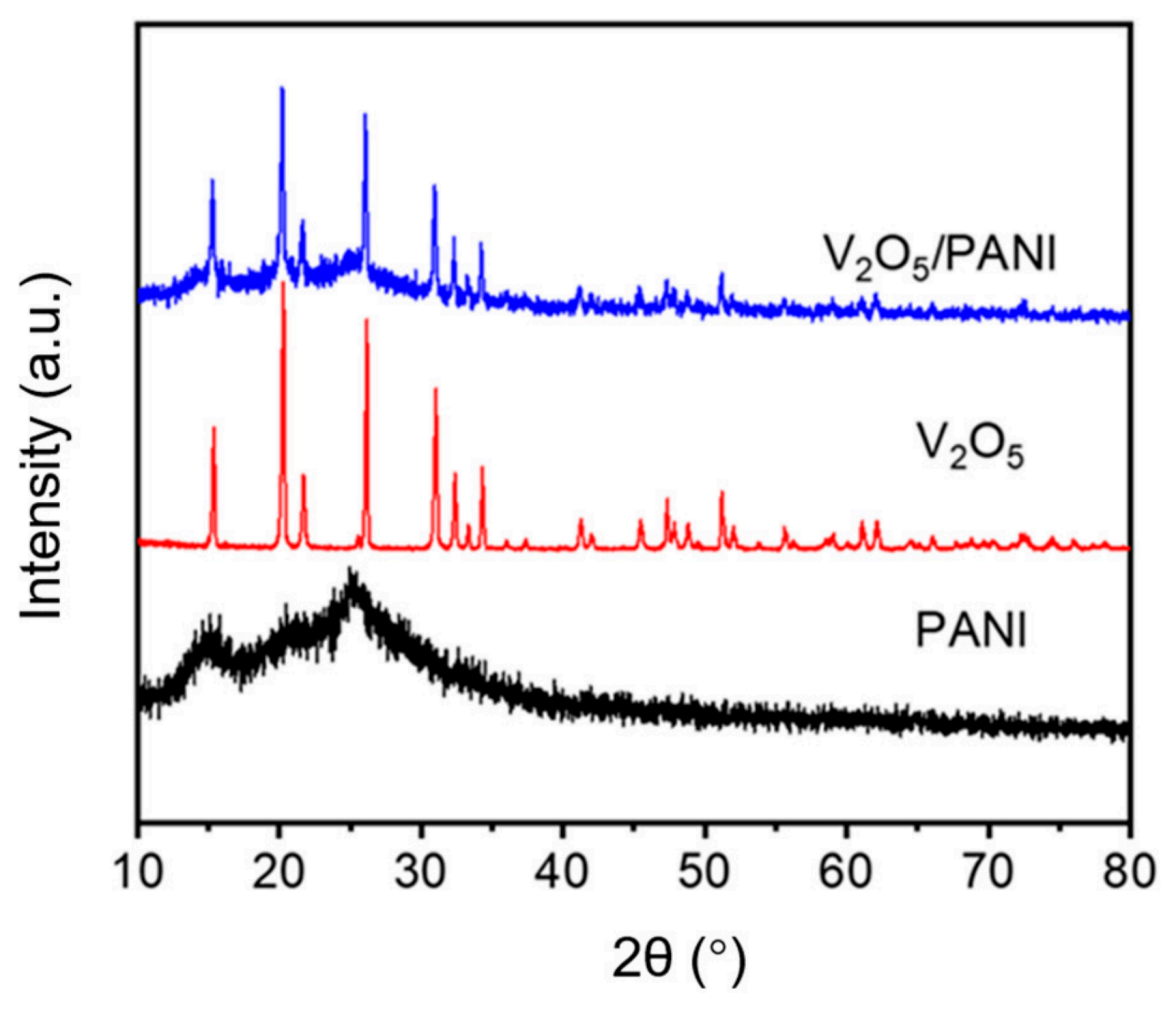
XRD patterns of V_2_O_5_, PANI, and V_2_O_5_/PANI composites.

XRD tests were performed on pure V_2_O_5_, pure PANI, and V_2_O_5_/PANI composites to clarify the phase composition and crystal structure characteristics of the samples, and the corresponding diffraction patterns are presented in Figure 3. All characteristic diffraction peaks of the as-prepared pure V_2_O_5_ are fully consistent with the standard card (PDF#01-086-2248) of orthorhombic V_2_O_5_. No impurity diffraction signals are observed in the whole spectrum, verifying the excellent phase purity of the synthesized sheet-like V_2_O_5_. Meanwhile, the sharp diffraction peaks with a narrow full width at half-maximum confirm that the V_2_O_5_ nanosheets possess a regular lattice arrangement, complete crystal structure, and favorable crystallinity. Different from crystalline V_2_O_5_, the XRD pattern of pure PANI only shows weak and broad diffuse diffraction humps without distinct sharp characteristic peaks, which corresponds to the typical amorphous structural characteristics of polyaniline polymers. For the V_2_O_5_/PANI composite, the XRD pattern completely retains all characteristic diffraction peaks of pure V_2_O_5_. No new diffraction peaks are generated, and no shift, attenuation, or disappearance of V_2_O_5_ characteristic peaks is observed. These results sufficiently indicate that the intrinsic orthorhombic crystal structure of V_2_O_5_ is well preserved without obvious lattice distortion during the PANI composite modification process, and a structurally stable hierarchical V_2_O_5_/PANI composite structure is successfully constructed. Crucially, no obvious shift in the characteristic diffraction peaks is observed, confirming that the combination of V_2_O_5_ and PANI primarily involves physical intermixing rather than chemical lattice incorporation. Therefore, the dramatic performance enhancement in the PAV20 composite cannot be attributed to bulk phase modifications but rather must originate from synergistic effects in morphological integration and interfacial band alignment.

**Figure 4:**
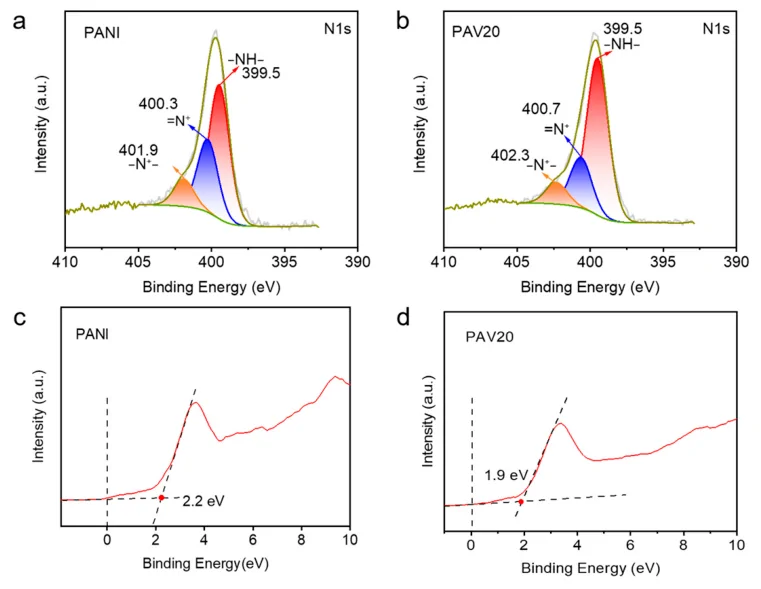
N 1s XPS spectra of (**a**) PANI and (**b**) PAV20, and valence band spectra of (**c**) PANI and (**d**) PAV20.

To probe the electronic structure and interfacial charge redistribution in the V_2_O_5_/PANI composite, high-resolution XPS characterizations were conducted on pure PANI and PAV20. Figure 4a,b show the N 1s spectra of pristine PANI and PAV20, respectively. Each spectrum is deconvoluted into neutral −NH− at ~399.5 eV [33] and two protonated nitrogen species (=N^+^ and −N^+^−) located within the 400–403 eV range [34]. Compared with pure PANI, both protonated nitrogen peaks of PAV20 display uniform positive shifts in binding energy: the =N^+^ peak shifts from 400.3 to 400.7 eV (+0.4 eV), and the −N^+^− peak shifts from 401.9 to 402.3 eV (+0.4 eV). In contrast, the neutral −NH− peak remains fixed at 399.5 eV for both samples, which is consistent with selective electron depletion at protonated nitrogen sites upon heterojunction formation. Valence band XPS spectra were further acquired to characterize bulk band alignment (Figure 4c,d). The VBM position, determined by the tangent extrapolation method, shifts to a lower binding energy from 2.2 eV (pristine PANI) to 1.9 eV (PAV20), corresponding to a 0.3 eV offset to the Fermi level. Collectively, the positive shifts of both protonated nitrogen peaks and the VBM offset provide direct experimental evidence that V_2_O_5_ acts as an electron acceptor and extracts electron density from PANI chains at the heterointerfaces during in situ polymerization.

### 3.2. Sensor Performance Test

**Figure 5:**
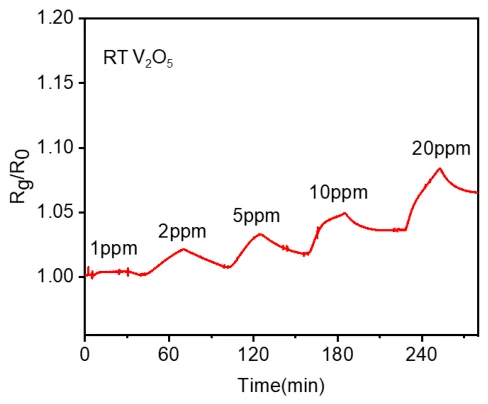
Dynamic response curve of V_2_O_5_ at room temperature.

To systematically investigate the room-temperature ammonia-sensing performance of the as-prepared material, gas-sensing measurements of the pristine V_2_O_5_-based sensor for NH_3_ were carried out at ambient temperature, and its response behavior to NH_3_ in the concentration range of 1–20 ppm was comprehensively characterized. The corresponding test results are presented in Figure 5. The pristine V_2_O_5_ sensor exhibits an intrinsic sensing capability for NH_3_ at room temperature; the device shows a baseline resistance shift upon NH_3_ exposure and partial recovery when subsequently purged with ambient air. A remarkable increase in resistance is observed once the sensor is exposed to NH_3_, while the resistance gradually returns to its baseline value after the removal of the target gas and re-exposure to air, demonstrating the typical characteristics of a p-type semiconductor. It is well established that V_2_O_5_ belongs to the classic n-type metal oxide semiconductors [35]. In an air atmosphere, adsorbed oxygen and water molecules act as electron acceptors and bend the surface energy band upward, thereby boosting electrical conductivity. Upon the introduction of NH_3_, the alkaline NH_3_ molecules interact with the surface-adsorbed species (including water-derived hydroxyl groups and protonated surface sites), which neutralizes the surface hole accumulation layer and thereby reduces the effective carrier concentration, increasing the sensor resistance. Such anomalous p-type-like behavior has been previously reported for V_2_O_5_ nanostructures and is attributed to surface adsorption effects involving water molecules rather than bulk conductivity type reversal [36,37]. As continuous NH_3_ injection further depletes the surface adsorbates, the surface energy band bends downward, ultimately leading to a prominent rise in sensor resistance.

**Figure 6:**
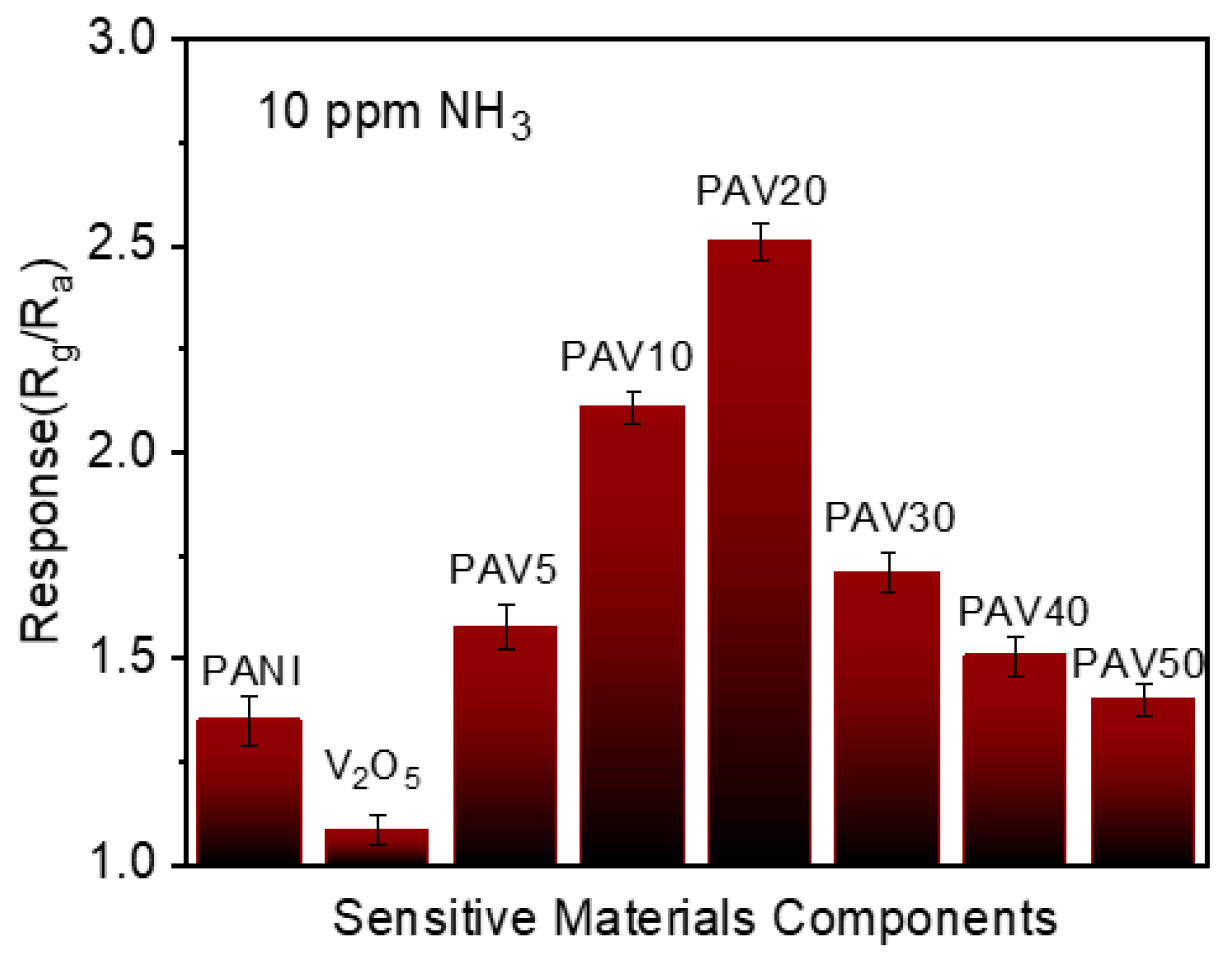
Response of sensitive materials with different molar ratios to 10 ppm NH_3_.

Although pristine V_2_O_5_ exhibits NH_3_-sensing activity at ambient temperature, its response magnitude is relatively low (Figure 6), and the sensor shows incomplete baseline recovery after gas exposure—a common challenge for semiconductor gas sensors operating at room temperature [38,39]. To overcome the limited sensitivity of single-component V_2_O_5_, PANI, known for its effective ammonia recognition and favorable electrical conductivity, was integrated with V_2_O_5_ to synthesize V_2_O_5_/PANI hybrid materials aimed at substantially enhancing the sensing performance. Room-temperature NH_3_-sensing measurements were then performed on V_2_O_5_/PANI hybrids with various V_2_O_5_ loading fractions, and the results are displayed in Figure 6, with each test repeated three times. Both the pristine V_2_O_5_ and pure PANI sensors obtain marginal response signals for NH_3_ under ambient conditions. In striking contrast, the V_2_O_5_/PANI composites exhibit remarkably enhanced sensing performance. The response magnitude rises first and then declines with increasing V_2_O_5_ loading, among which the PAV20 sample with 20% V_2_O_5_ achieves the optimal ammonia-sensing capability. Such performance evolution can be rationalized by the material microstructural features and synergistic interfacial effects of the two components. At moderate doping ratios, V_2_O_5_ nanosheets are homogeneously dispersed within the PANI matrix, establishing intimate contact between the two phases and generating prominent sensing synergy. Meanwhile, the hybrid architecture manipulates the pore configuration and elevates the specific surface area, providing abundant active sites for ammonia adsorption and drastically boosting the material’s capture capacity and response to NH_3_. Nevertheless, excessive V_2_O_5_ loadings beyond 20% trigger severe particle agglomeration and stacking, disrupting the continuous fibrous network of PANI. This agglomeration drastically reduces the effective specific surface area and depletes surface adsorption sites. Furthermore, the densely aggregated aggregates impede gas diffusion throughout the sensing layer, leading to a gradual deterioration of the overall sensing response.

The baseline resistances of the sensors at room temperature are approximately 30 kΩ for pure PANI, 7 MΩ for PAV20, and 70 MΩ for pure V_2_O_5_, following the order PANI < PAV20 < V_2_O_5_. Pure V_2_O_5_ shows extremely high resistance, owing to its wide bandgap and sparse intrinsic carriers, while interwoven PANI nanofibers construct continuous conductive pathways and present the lowest resistance. The moderate resistance of PAV20 is consistent with the formation of a V_2_O_5_/PANI heterojunction that modulates charge transport in the composite.

**Figure 7:**
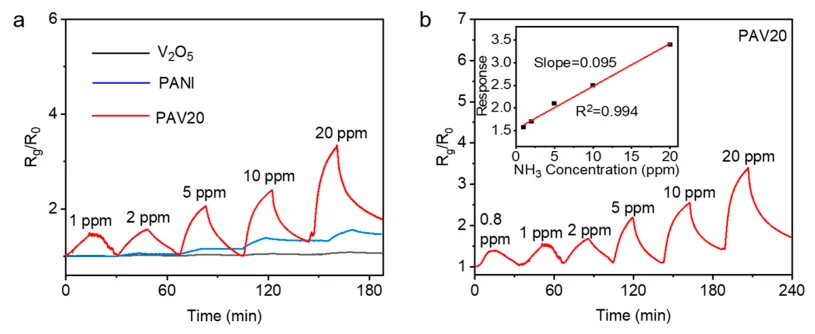
(**a**) Dynamic response curve of the sensor based on V_2_O_5_, PANI, and PAV20 composite structure to NH_3_. (**b**) The dynamic response and linear fitting of the sensor based on PAV20 composite structure.

Figure 7a compares the dynamic response curves of pristine V_2_O_5_, pure PANI, and PAV20 composite sensors to NH_3_ ranging from 1 to 20 ppm at room temperature. The dynamic curves visually reveal that both single-component counterparts deliver feeble response signals and insufficient sensitivity, failing to meet the requirements for ammonia detection under complex background atmospheres. In contrast, the PAV20 composite with the optimal loading ratio exhibits a drastically higher response magnitude than both pure materials, which strongly verifies that the construction of hierarchical V_2_O_5_/PANI heterostructures constitutes an effective strategy to boost room-temperature NH_3_-sensing performance and overcome the inherent drawbacks of single-phase sensing materials. Notably, although the recovery kinetics at ambient temperature are inherently more gradual than those of conventional high-temperature MOS sensors, the PAV20 sensor’s resistance returns to its baseline upon exposure to ambient air without any external thermal or UV assistance. Considering the target deployment in flammable chemical parks where heating elements are strictly prohibited, this self-sustained room-temperature recovery characteristic is practically favorable for intermittent leakage monitoring scenarios. On this basis, a comprehensive investigation into the full-range sensing performance of the PAV20 sensor was further carried out. Figure 7b presents the response versus NH_3_ concentration for the PAV20 sensor over 0.8–20 ppm. The inset shows the linear fitting in the 1–20 ppm range (R^2^ = 0.994), confirming the outstanding linear sensing behavior of the composite sensor. Based on this linear response, the limit of detection (LOD) was calculated using the equation LOD = 3σ/S, where σ represents the standard deviation of the system baseline noise, and S denotes the slope of the linear portion of the response curve [40]. The σ value was determined to be 1.37 × 10^−4^, and S = 0.095, yielding an LOD of approximately 4.3 ppb. This value is among the lowest reported for room-temperature PANI-based NH_3_ sensors, further confirming the potential of the PAV20 composite for trace ammonia detection. The response and recovery times, defined as 90% of the full magnitude change in the gas response factor, were approximately 540 s and 840 s for the PAV20 sensor to 10 ppm NH_3_ at room temperature.

**Figure 8:**
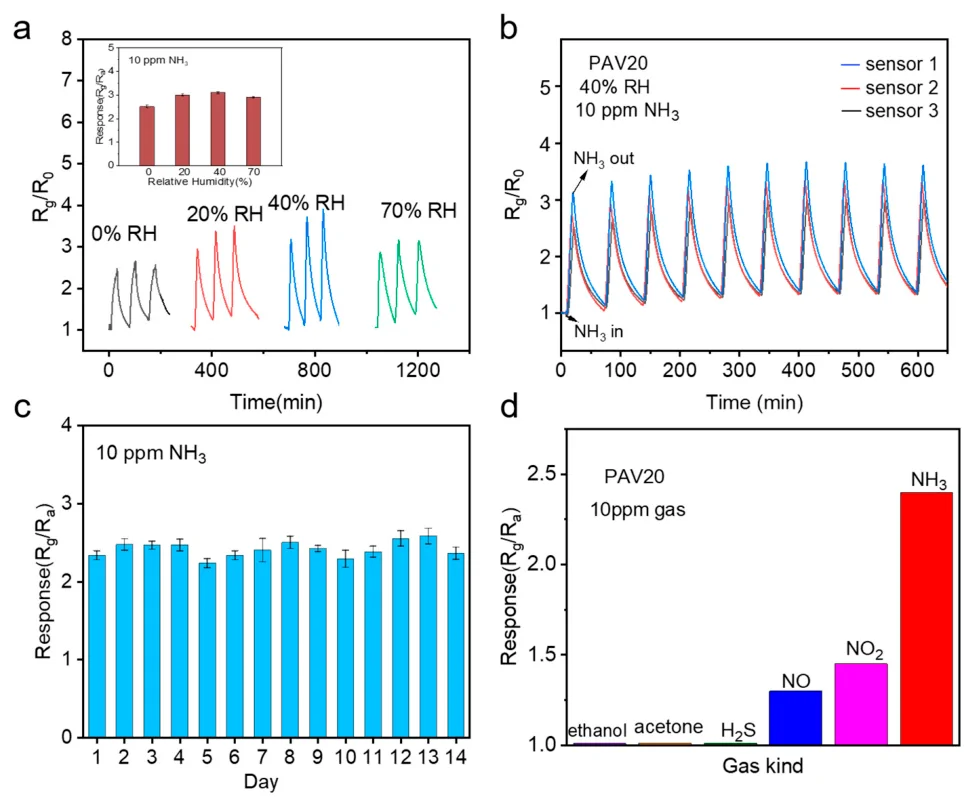
(**a**) Dynamic response–recovery curves and corresponding average sensing response bar chart (inset) of the PAV20 sensor to 10 ppm NH_3_ at 0%, 20%, 40%, and 70% relative humidity. (**b**) The response of three different independent sensors to 10 cycles of 10 ppm NH_3_ at 40% RH. (**c**) Stability test to 10 ppm NH_3_. (**d**) Selectivity to 10 ppm of different gases for the sensor based on PAV20 composite material at room temperature.

In addition to evaluating the gas-sensing performance, the humidity-dependent characteristics of the PAV20 sensor were systematically investigated. As shown in Figure 8a, the main panel presents complete dynamic response and recovery cycles to 10 ppm NH_3_ under four distinct relative humidity (RH) conditions: 0%, 20%, 40%, and 70%. The inset bar chart quantitatively depicts the average response magnitude at each humidity level. Notably, the sensor response initially increases from 0% to 40% RH, followed by a slight decline at 70% RH; however, the response at 70% RH remains elevated relative to that at 0% RH. More importantly, the recovery behavior improves markedly with increasing humidity. At 0% RH, the sensor exhibits incomplete recovery with a pronounced baseline drift, whereas under humid conditions (20–70% RH), the resistance moves significantly closer to the initial baseline between consecutive cycles. This non-monotonic trend indicates a competitive interplay between two simultaneous mechanisms: at lower humidity levels (0–40% RH), water molecules enhance NH_3_ detection by inducing swelling in the PANI chains and facilitating additional proton transfer pathways, as well as promoting the desorption of adsorbed NH_3_ species; conversely, at higher humidity levels (40–70% RH), excess H_2_O molecules compete with NH_3_ for active adsorption sites, partially inhibiting the sensor response [41,42]. The enhanced recovery under humid conditions can be attributed to the plasticizing effect of water molecules on the PANI component, which facilitates the re-protonation process after NH_3_ desorption, and to the general promoting effect of adsorbed water on the desorption kinetics of surface-adsorbed species, a phenomenon widely observed in metal oxide semiconductor gas sensors operating under humid conditions [43]. In addition to the response magnitude and recovery behavior, the baseline resistance of the PAV20 sensor is notably affected by humidity. The baseline resistance decreases by approximately 53%, 58%, and 67% at 20% RH, 40% RH, and 70% RH, respectively. Upon returning to dry conditions, the baseline resistance recovers to its original level, confirming the reversibility of the humidity-induced resistance change. This monotonic decrease is attributed to adsorbed water molecules that participate in proton exchange with the imine nitrogen sites of PANI, effectively increasing the carrier concentration. Despite the significant baseline shift, the sensor maintains a distinguishable and reproducible response to NH_3_ across all tested humidity levels, confirming that the acid–base interaction between NH_3_ and PANI remains the dominant sensing mechanism [44].

Furthermore, the practical utility of the PAV20 sensor was thoroughly evaluated in terms of cyclic reproducibility, long-term stability, and selectivity. As shown in Figure 8b, ten consecutive dynamic response cycles were recorded at 40% RH for three independently fabricated PAV20 sensors upon exposure to 10 ppm NH_3_. The resistance increases clearly upon each NH_3_ exposure and recovers upon gas removal, confirming reproducible sensing behavior. All three sensors exhibit consistent response trends across the ten cycles with a relative standard deviation (RSD) of 1.92%, demonstrating good cycle-to-cycle and device-to-device reproducibility. A minor residual baseline drift is observed over consecutive cycles, consistent with the PANI-based sensors at ambient temperature and attributed to the slow desorption kinetics of NH_3_ [45]. Importantly, the response ratio Rg/Ra remains consistent throughout all cycles, indicating that the sensor’s sensitivity is minimally affected by baseline drift and that the response amplitudes are genuinely reproducible. Figure 8c presents the results of a 14-day stability test, during which the response to 10 ppm NH_3_ was measured periodically. The response values exhibit only slight variations over the entire period, with an RSD of less than 4.1%, demonstrating the stable and reliable sensing performance of the PAV20 composite under normal storage conditions. Figure 8d exhibits the response values of the sensor to various gases at a concentration of 10 ppm under room-temperature conditions. The results reveal that the sensor achieves the maximum response to NH_3_. Negligible responses are observed for acetone and ethanol at the same concentration, while only weak signals are obtained for nitrogen oxides (NO_2_ and NO).

## 4. Discussion

To comprehensively evaluate the room-temperature ammonia (NH_3_)-sensing performance of the developed 20% V_2_O_5_/PANI composite film, Table 1 summarizes the sensing properties of polyaniline–metal oxide composite systems reported in recent years. Compared with other PANI-based room-temperature NH_3_ sensors, the 20% V_2_O_5_/PANI film in this work delivers a higher response at 10 ppm NH_3_ and an ultralow LOD (4.3 ppb) while maintaining a simple composition and straightforward fabrication process. These advantages highlight its great potential for practical detection of low-concentration ammonia at room temperature.

As concluded from the XRD analysis, the absence of lattice distortion or new phase formation unequivocally excludes the possibility of bulk-doping effects. Consequently, the extraordinary sensing performance of the PAV20 composite must be governed by the interfacial charge transfer and morphological synergy.

The resistance variation of sensing films is jointly determined by the redox properties of target gases and the carrier transport behavior within sensitive materials. The overall resistance of composite films rises after exposure to NH_3_, exhibiting a hole-dominated sensing response. The NH_3_-sensing mechanism of pure V_2_O_5_ nanostructures was elaborated in the previous section and will not be repeated here.

The gas-sensing behavior of pure PANI is governed by the reversible equilibrium between protonation and deprotonation. Proton-doped emeraldine salt (ES) presents excellent electrical conductivity, while deprotonation of polymer chains converts it into insulating emeraldine base (EB). The doping state of polyaniline can be reversibly tuned by acid–base atmospheres, as illustrated in Equation (1):

(1) $$\mathrm{PANI}\;-\;H^{+}(\mathrm{ES})\;+\;{\mathrm{NH}}_{3}\;\to \;\mathrm{PANI}(\mathrm{EB})\;+\;{NH}_{4}^{+}$$

When the sensitive film is exposed to NH_3_ atmosphere, alkaline NH_3_ captures protons from polyaniline chains and drives the transition from conductive ES to insulating EB. This process reduces the quantity of mobile holes, decreases the film conductivity, and elevates the overall resistance of the sensing layer. After removing NH_3_ and exposing the film to ambient air, the adsorbed NH_4_^+^ spontaneously dissociates and releases protons to re-dope polyaniline, recovering the electrical conductivity and returning the resistance to its initial level.

The remarkable enhancement in the sensing performance of the PAV20 composites originates from the synergistic effect of hierarchical porous microstructures and bi-phase heterogeneous interfaces, which can be discussed from two perspectives. On the one hand, the hierarchical porous microstructure enhances gas adsorption capacity. In situ polymerized PANI fibers uniformly coat and interpenetrate the surface of V_2_O_5_ nanostructures. This architecture effectively restrains the self-aggregation of polyaniline and constructs a loose, hierarchical porous network. Such a well-interpenetrated configuration not only shortens the gas diffusion pathways within the sensing layer but also ensures a high exposure frequency of the dual-component interfaces to the incoming NH_3_ molecules, thereby promoting sufficient interfacial interactions.

**Figure 9:**
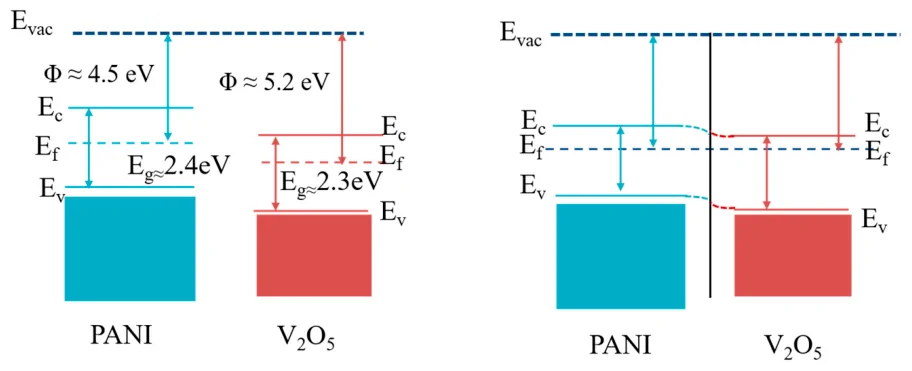
Energy band diagrams of V_2_O_5_-PANI.

After forming intimate heterostructure contact between V_2_O_5_ and PANI, spontaneous charge rearrangement occurs, which is driven by the mismatch of the intrinsic work functions of the two materials. On the other hand, the interfacial charge redistribution at the dual-component boundary plays a crucial role in enhancing the sensing performance. The intimate contact between the V_2_O_5_ nanostructures and PANI constructs a coupled dual-component interface, and the corresponding vacuum level, conduction band minimum, valence band maximum, and Fermi level are marked in Figure 9. The band structure parameters used to construct Figure 9 are derived from previously reported experimental characterization results [51,52,53]. More importantly, the interfacial charge redistribution and band bending behavior proposed in the energy-level diagram are directly verified by our XPS valence band (VB) spectra. The valence band maximum positions of pure PANI and the PAV20 composite were extracted via tangent extrapolation of VB-XPS curves. Compared with pristine PANI, the VBM of the PANI component within PAV20 shifts toward a lower binding energy. This obvious shift of the valence band edge confirms directional band bending at the dual-component interface, consistent with the charge redistribution trend driven by work function mismatch. This valence band shift, together with the positive binding energy shifts of protonated nitrogen in the N 1s XPS spectra, directly verifies the electron transfer behavior across the heterostructure interface and leads to interfacial charge redistribution within the composite.

ES features abundant mobile charge carriers, whereas pure V_2_O_5_ exhibits a much lower intrinsic concentration of free charge carriers. Driven by the discrepancy in intrinsic work functions between the two materials, spontaneous charge redistribution occurs across the tight contact interface to align their Fermi levels under thermal equilibrium, as visualized in Figure 9. This charge rearrangement induces carrier depletion on the PANI side adjacent to the two-phase boundary. This charge loss on the PANI surface is offset by accumulated carriers on the adjacent V_2_O_5_ surface to maintain interfacial electrical neutrality. The formation of this interfacial depletion region reduces the overall quantity of movable charge carriers in the composite system, thereby elevating the baseline resistance of the PAV20 films relative to that of pure PANI. Upon NH_3_ injection, the concentration of mobile charge carriers within PANI further declines, which further diminishes the carrier density at the bi-phase interface. The charge transport resistance rises significantly, which greatly magnifies the resistance variation and endows the composite with a superior sensing response to NH_3_.

Furthermore, the interconnected porous structure and stable two-phase interface not only enhance the intrinsic sensing response to NH_3_ but also mitigate the adverse effects of humidity interference, incomplete NH_3_ desorption at room temperature, and long-term material degradation. Additionally, the unique proton exchange interaction between alkaline NH_3_ and PANI chains enables the composite to achieve excellent selective recognition of ammonia over various interfering gases.

The room-temperature operation of the PAV20 sensor represents a deliberate design choice for low-power applications. While elevated operating temperatures would improve recovery kinetics and reduce baseline drift, they would also increase power consumption and limit the sensor’s applicability in portable and wearable devices. The present results demonstrate that acceptable sensing performance—including a reproducible response magnitude over multiple cycles—can be achieved at room temperature, with the minor baseline drift manageable through periodic recalibration.

## 5. Conclusions

In this work, structurally controllable V_2_O_5_/PANI hierarchical porous composites were synthesized via a straightforward coprecipitation approach combined with in situ polymerization. A systematic investigation was conducted to elucidate the relationship among the component ratio, microscopic morphology, and room-temperature NH_3_-sensing performance. Structural analyses reveal that interlaced PANI fiber networks and V_2_O_5_ nanoparticles assemble into a loosely packed hierarchical porous framework. This dual-phase synergistic interaction effectively inhibits material agglomeration, increases the density of surface active sites, and optimizes gas diffusion dynamics, thereby enhancing the gas utilization efficiency of the sensing layer. However, excessive oxide doping compromises structural integrity and induces aggregation defects, resulting in diminished sensing capabilities. Gas-sensing evaluations demonstrate that the composite containing 20% V_2_O_5_ achieves optimal room-temperature sensing performance, with a response value of 2.5 to 10 ppm NH_3_, which is 2.0 to 2.5 times greater than that of the pure constituent materials. The sensor reliably detects ammonia across a relative humidity range of 0% to 70%, demonstrating moderate resistance to humidity levels commonly encountered in real-world environments. Additionally, the composite sensor exhibits excellent selectivity for NH_3_, even when exposed to various interfering gases at equal concentrations. Compared to recently developed polyaniline–metal oxide sensing systems, the prepared composite delivers competitive and comprehensive sensing performance. This enhanced sensing capability results from the combined effects of structural design and interfacial modulation. Specifically, the hierarchical porous structure provides numerous sites for gas interaction, while the heterogeneous band alignment effectively manages carrier transport and amplifies resistance changes. This polymer–oxide hierarchical composite strategy achieves dual synergistic enhancements by leveraging both structural advantages and interfacial effects, offering a simple and effective design approach for high-performance gas sensors operating at room temperature. Due to its straightforward fabrication, good humidity tolerance under ambient conditions, and strong sensitivity to trace ammonia, the V_2_O_5_/PANI sensor holds significant potential for miniaturization and integration. It is especially well suited for UAV-based mobile detection systems aimed at intelligent, large-scale monitoring of ammonia leaks in chemical industrial parks, providing valuable applications in low-power, smart industrial gas safety monitoring. Film thickness and microstructural porosity can be further regulated to shorten the response and recovery times of the sensor in subsequent research.

## Figures and Tables

**Table 1:** Comparison of the performance of polyaniline–metal oxide-based NH_3_ sensors reported in the literature.

Material	Working Temperature	Response	Response/Recovery Time	LOD	Ref.
V_2_O_5_/PANI/1 wt%GO NCPs	RT	~1.3 (10 ppm)	70 s/520 s	0.5 ppm	[33]
PANI/SnO_2_	RT	1.4 (10 ppm)	78 s/102 s	/	[46]
RGO-PANI	RT	3.5 (100 ppm)	97 s/680 s	5 ppm	[47]
TiO_2_/PANI/GO	RT	1.96 (100 ppm)	169 s/1516 s	2.14 ppm	[48]
PANI/Ti_3_C_2_T_X_/TiO_2_	RT	2.3 (10 ppm)	266 s/342 s	0.02 ppm	[49]
h-NiO-PANI	RT	1.43 (10 ppm)	149 s/257 s	/	[50]
20%V_2_O_5_/PANI	RT	2.5 (10 ppm)	540 s/840 s	~0.004 ppm	This work

*Note: All response values are converted to R_g_/R_a_ or R_a_/R_g_ for direct comparison*.

## Data Availability

The data used for this study are available in the manuscript.

## References

[1] Kwak D., Lei Y., Maric R. (2019). Ammonia gas sensors: A comprehensive review. Talanta.

[2] Nadargi D.Y., Umar A., Nadargi J.D., Lokare S.A., Akbar S., Mulla I.S., Suryavanshi S.S., Bhandari N.L., Chaskar M.G. (2023). Gas sensors and factors influencing sensing mechanism with a special focus on MOS sensors. J. Mater. Sci..

[3] Xue S., Cao S., Huang Z., Yang D., Zhang G. (2021). Improving Gas-Sensing Performance Based on MOS Nanomaterials: A Review. Materials.

[4] Ou L.-X., Liu M.-Y., Zhu L.-Y., Zhang D.W., Lu H.-L. (2022). Recent Progress on Flexible Room-Temperature Gas Sensors Based on Metal Oxide Semiconductor. Nano-Micro Lett..

[5] Eranna G., Joshi B.C., Runthala D.P., Gupta R.P. (2010). Oxide Materials for Development of Integrated Gas Sensors—A Comprehensive Review. Crit. Rev. Solid State Mater. Sci..

[6] Batzill M., Diebold U. (2007). Surface studies of gas sensing metal oxides. Phys. Chem. Chem. Phys..

[7] Tian M., Wu Y., Zhao Z., Chen Z., Zhu N., Jiang X. (2026). Materials design strategies for semiconducting metal-oxide chemiresistive gas sensors: A review. RSC Appl. Interfaces.

[8] Muhammad P., Zada A., Rashid J., Hanif S., Gao Y., Li C., Li Y., Fan K., Wang Y. (2024). Defect Engineering in Nanocatalysts: From Design and Synthesis to Applications. Adv. Funct. Mater..

[9] Huang J., Wan Q. (2009). Gas Sensors Based on Semiconducting Metal Oxide One-Dimensional Nanostructures. Sensors.

[10] Gurlo A. (2011). Nanosensors: Towards morphological control of gas sensing activity. SnO_2_, In_2_O_3_, ZnO and WO_3_ case studies. Nanoscale.

[11] Liu L., Wang Y., Liu Y., Wang S., Li T., Feng S., Qin S., Zhang T. (2022). Heteronanostructural metal oxide-based gas microsensors. Microsyst. Nanoeng..

[12] Zhang C., Qian L., Zeng W. (2025). MOS based gas sensor in detection of volatile organic compounds: A review. Sens. Actuators A Phys..

[13] Barsan N., Koziej D., Weimar U. (2007). Metal oxide-based gas sensor research: How to?. Sens. Actuators B Chem..

[14] Shen X., Dhar S., Pantelides S.T. (2015). Atomic origin of high-temperature electron trapping in metal-oxide-semiconductor devices. Appl. Phys. Lett..

[15] Amiri V., Roshan H., Mirzaei A., Sheikhi M.H. (2020). A Review of Nanostructured Resistive-Based Vanadium Oxide Gas Sensors. Chemosensors.

[16] Yin H., Song C., Wang Z., Shao H., Li Y., Deng H., Ma Q., Yu K. (2019). Self-Assembled Vanadium Oxide Nanoflakes for p-Type Ammonia Sensors at Room Temperature. Nanomaterials.

[17] Mounasamy V., Mani G.K., Tsuchiya K., Madanagurusamy S. (2021). Nanoimprint assisted free standing porous vanadium oxide nanosheet based ammonia sensor. Appl. Surf. Sci..

[18] Amarnath M., Gurunathan K. (2021). Selective ammonia sensing response of vanadium doped cerium oxide nanorods wrapped reduced graphene oxide electrodes at room temperature. Sens. Actuators B Chem..

[19] Sucharitakul S., Ye G., Lambrecht W.R.L., Bhandari C., Gross A., He R., Poelman H., Gao X.P.A. (2017). V_2_O_5_: A 2D van der Waals Oxide with Strong In-Plane Electrical and Optical Anisotropy. ACS Appl. Mater. Interfaces.

[20] Chackrabarti S., Zargar R.A. (2026). Review: Vanadium pentoxide thin films-advanced doping strategies and interface engineering for next-generation applications. J. Mater. Sci..

[21] Wang J., Tan X., Xu X., Chen D., Ren Z., Zhang Z., Jiang H., Meng C., Zhang Y. (2026). Dual storage mechanism via hydrogen bonding and redox in 1D MOF-pillared vanadium oxide for high-performance aqueous ammonium-ion batteries. J. Colloid Interface Sci..

[22] Shi H.B.a.G. (2007). Gas Sensors Based on Conducting Polymers. Sensors.

[23] Nijkoops A., Ciocca M., Aurora Costa Angeli M., Ahmad M., Boom R., Münzenrieder N., Lugli P., Petti L. (2025). Recent advances in conducting polymer chemiresistive sensors for ammonia gas detection: Materials, characterization, and applications. J. Phys. Mater..

[24] Le T.-H., Kim Y., Yoon H. (2017). Electrical and Electrochemical Properties of Conducting Polymers. Polymers.

[25] Nulimu A., Aldongarov A., Sarsenova S., Ibrayeva A., Karibayev M. (2025). Unraveling the Role of Functional Groups in Polyaniline for NH_3_ Sensing: A Theoretical Approach. Eng. Sci..

[26] Liu C., Tai H., Zhang P., Yuan Z., Du X., Xie G., Jiang Y. (2018). A high-performance flexible gas sensor based on self-assembled PANI-CeO_2_ nanocomposite thin film for trace-level NH_3_ detection at room temperature. Sens. Actuators B Chem..

[27] Tai H., Jiang Y., Xie G., Yu J., Chen X., Ying Z. (2008). Influence of polymerization temperature on NH_3_ response of PANI/TiO_2_ thin film gas sensor. Sens. Actuators B Chem..

[28] Lee C.-T., Wang Y.-S. (2019). High-performance room temperature NH_3_ gas sensors based on polyaniline-reduced graphene oxide nanocomposite sensitive membrane. J. Alloys Compd..

[29] Mathew M., Shinde P.V., Samal R., Rout C.S. (2021). A review on mechanisms and recent developments in p-n heterojunctions of 2D materials for gas sensing applications. J. Mater. Sci..

[30] Zhu Y., Liu X., Hu X., Wang T., Parkin I.P., Wang M., Boruah B.D. (2024). Polyaniline and water pre-intercalated V_2_O_5_ cathodes for high-performance planar zinc-ion micro-batteries. Chem. Eng. J..

[31] Bi W., Wang J., Jahrman E.P., Seidler G.T., Gao G., Wu G., Cao G. (2019). Interface Engineering V_2_O_5_ Nanofibers for High-Energy and Durable Supercapacitors. Small.

[32] Chen C., Zhou X., Lv C., Ke X., Chen L., Liu X. (2025). Design and application of an integrated stretchable energy-storage gas sensing system with different morphologies of PANI@V_2_O_5_. Chem. Eng. J..

[33] Xing X., Du L., Feng D., Wang C., Tian Y., Li Z., Liu H., Yang D. (2022). Twistable and tailorable V_2_O_5_/PANI/GO nanocomposites textile for wearable ammonia sensing. Sens. Actuators B Chem..

[34] Raorane C.J., Kim S.-C. (2026). Interfacial Engineering of V_2_O_5_ via Conductive Polyaniline for Accelerated Hydrogen Evolution Reaction. Polymers.

[35] Schneider K., Lubecka M., Czapla A. (2016). V_2_O_5_ thin films for gas sensor applications. Sens. Actuators B Chem..

[36] Van Duy L., Nguyet T.T., Le D.T.T., Van Duy N., Nguyen H., Biasioli F., Tonezzer M., Di Natale C., Hoa N.D. (2022). Room Temperature Ammonia Gas Sensor Based on p-Type-like V_2_O_5_ Nanosheets towards Food Spoilage Monitoring. Nanomaterials.

[37] Benkahoul M., Zayed M.K., Solieman A., Alamri S.N. (2017). Spray deposition of V_4_O_9_ and V_2_O_5_ thin films and post-annealing formation of thermochromic VO_2_. J. Alloys Compd..

[38] Qin Y., Wang Z., Zou Y., Yang W. (2026). Memristive Ammonia Gas Sensor Based on CuO Nanosheets/ZnO Nanoparticles Heterostructures with Pressure Assisted Recovery. ACS Appl. Nano Mater..

[39] Vasiliev R., Kurtina D., Udalova N., Platonov V., Nasriddinov A., Shatalova T., Novotortsev R., Li X., Rumyantseva M. (2022). SnS_2_ Nanosheets as a Template for 2D SnO_2_ Sensitive Material: Nanostructure and Surface Composition Effects. Materials.

[40] Freddi S., Carro P., Creus A.H., Sangaletti L., Rodriguez Gonzalez M.C. (2025). Selective enhancement of graphene ammonia sensing by electrochemical palladium nanoparticle decoration: Ab initio insights into the sensing response. Nanoscale.

[41] Wang Z., Lan K., Wang Z., Wei J., Chen R., Qin G. (2025). High-performance PANI sensor on silicon nanowire arrays for sub-ppb NH_3_ detection. Talanta.

[42] Abdulla S., Mathew T.L., Pullithadathil B. (2015). Highly sensitive, room temperature gas sensor based on polyaniline-multiwalled carbon nanotubes (PANI/MWCNTs) nanocomposite for trace-level ammonia detection. Sens. Actuators B Chem..

[43] Santos-Ceballos J.C., Salehnia F., Güell F., Romero A., Vilanova X., Llobet E. (2024). Room-Temperature Ammonia Sensing Using Polyaniline-Coated Laser-Induced Graphene. Sensors.

[44] Kroutil J., Laposa A., Ahmad A., Voves J., Povolny V., Klimsa L., Davydova M., Husak M. (2022). A chemiresistive sensor array based on polyaniline nanocomposites and machine learning classification. Beilstein J. Nanotechnol..

[45] Pan Y., Li X., Shi H., Du J., Fan S., Cao M., Li S., Liu T. (2025). High-performance flexible NH_3_ sensor based on PANI/Ti_3_C_2_T_x_ composites for real-time fish spoilage detection. Chem. Eng. J..

[46] Chen Y., Fu H., Bai Y., Yang X., Xiong S., Han D., An X. (2024). Room-temperature NH_3_-sensor: SnO_2_@PANI core-shell hollow spheres. Sens. Actuators B Chem..

[47] Hadano F.S., Gavim A.E.X., Stefanelo J.C., Gusso S.L., Macedo A.G., Rodrigues P.C., Mohd Yusoff A.R.b., Schneider F.K., Deus J.F.d., José da Silva W. (2021). NH_3_ Sensor Based on rGO-PANI Composite with Improved Sensitivity. Sensors.

[48] Ragab H.M., Diab N.S., Aleid G.M., Aziz R.A., Farea M.O., Yusof N., Farea M.A. (2025). Enhanced detection of ammonia (NH_3_) using a TiO_2_/PANI/GO composite for real-time environmental monitoring. Chem. Phys. Lett..

[49] Xiong J., Cai Y., Nie X., Wang Y., Song H., Sharif H.M.A., Li Z., Li C. (2023). PANI/3D crumpled Ti_3_C_2_T_X_/TiO_2_ nanocomposites for flexible conductometric NH_3_ sensors working at room temperature. Sens. Actuators B Chem..

[50] Hu Q., Wang Z., Chang J., Wan P., Huang J., Feng L. (2021). Design and preparation of hollow NiO sphere- polyaniline composite for NH_3_ gas sensing at room temperature. Sens. Actuators B Chem..

[51] Lee S., Wang Q., Chakrapani V. (2024). Electronic vs optical bandgap of stoichiometric V_2_O_5_: Effects of surface transfer doping and electron accumulation layer. J. Appl. Phys..

[52] Abdulrazzaq O., Bourdo S.E., Saini V., Watanabe F., Barnes B., Ghosh A., Biris A.S. (2015). Tuning the work function of polyaniline via camphorsulfonic acid: An X-ray photoelectron spectroscopy investigation. RSC Adv..

[53] Karaoğlan N., Bindal C. (2018). Synthesis and optical characterization of benzene sulfonic acid doped polyaniline. Eng. Sci. Technol. Int. J..

